# Meetings of Societies

**Published:** 1906-03

**Authors:** 


### MEETINGS OF SOCIETIES.

### Bristol Medico-Chirurgical Society.

*December 13th, 1905.*

Mr. John Dacre, President, in the Chair.

Dr. P. Watson Williams showed the following cases: (*a*) A patient operated upon for double **frontal sinusitis** and **maxillary empyema.** (*b*) A case of **fibroma** of the left **vocal cord.** The frontal sinuses were dealt with by the Killian operation, and radical operation was performed on each of the maxillary antra with very successful results.

Dr. Bertram Rogers read notes on a case of **acute atrophy of the liver**, and showed sections and lantern slides illustrating the histology of the liver in the disease. (See p. 40.)

Dr. F. H. Edgeworth read a paper on a case of **acute yellow atrophy of the liver**, and showed specimens and lantern slides obtained from the case. (See p. 37.)

Dr. E. C. Williams read notes of a case of **splenomegalic hypertrophic biliary cirrhosis** in a child aged six years, and showed the patient. (See p. 42 of this issue of the *Journal*.) — Dr. Shingleton Smith alluded to a case of acute yellow atrophy of the liver he had seen in the Bristol Royal Infirmary seventeen or twenty years previously. The patient was a man with an alcoholic history, and he died in a comatose condition. The extraordinarily flabby condition of the liver had been characteristic. —Dr. Fortescue-Brickdale thought it would be better to name Dr. Rogers's case acute toxæmic jaundice. The weight of the liver had been normal for a child of that age.—Dr. Carey Coombs thought the atrophy described by Dr. Rogers was probably the terminal phase of a chronic process, as the boy had had a transient jaundice six months before death, and the liver was increased in size and density by proliferation of the connective tissue. The speaker quoted various cases to show that a scale of toxæmic jaundice could be constructed, progressing in intensity from biliary cirrhosis on the one hand to acute yellow atrophy on the other. These processes, which are similar in essence though differing in intensity, are analogous to those set up by phosphorus, the lesions of yellow fever, and other poisons. They are to be ascribed to poisonous bodies, probably bacteria and their products reaching the liver in the blood-stream, most likely by the portal vessels, and excreted by the bile. In their passage through the liver bile-ducts the hepatic parenchyma is damaged and inflammatory reaction in the connective tissue is set up. The liver has been shown to have the power of partially destroying the deleterious effect of bacterial toxins circulating through it; if this power be brought to bear upon the toxins causing toxæmic jaundice, the liver will suffer comparatively little, a certain degree of interstitial hyperplasia being the main histological result. This hyperplasia is gradually increased by repeated doses or a steady stream of the poison. Finally the immunising power of the liver is overwhelmed either by its having undergone gradual exhaustion or by the arrival of a larger dose of poison, and a rapid atrophy of the parenchyma occurs, bringing about a fatal result (as in Dr. Rogers's case). Dr. Edgeworth's patient, on the other hand, succumbed at once, presumably because the immunising power of her liver was too small or because the dose of poison was too large, and consequently the parenchyma of her liver rapidly underwent irreparable destruction. Hexner has shown that large single doses of ricin and abrin, inoculated into rabbits, produce cellular destruction, while smaller repeated doses of the same substances cause the formation of fibrous tissue and embryonic bile-ducts.—Dr Michell Clarke thought it might be well to keep the name acute atrophy for the more acute cases. He had published two cases of the kind in which the disease had followed cholera infantum in children, and these cases seemed to show more clearly than many the probable toxæmic nature of the disease. He would prefer to name Dr. Rogers's case one of acute cholæmia with necrosis of the liver. He was of opinion that implication of the kidneys played a large part in the final stages of the disease, and it was not until the kidney was attacked that the fatal coma ensued.—Dr. Rogers thought it was curious that the disease was not more common if it could be engendered by infantile diarrhœa. The overgrowth of fibrous tissue in the liver in this case had been slight.

Mr. T. Carwardine read notes on the following cases:—(*a*) **perforated duodenal ulcer**, (*b*) **perforated gastric ulcer**, (*c*) three successful **prostatectomies** (with specimen).—Mr. Kyle alluded to the possibility of troublesome symptoms following prostatectomy, and mentioned the trouble he had had in one case in getting the supra-pubic opening to close permanently. This result was finally obtained after dilating a stricture which had formed in the deep urethra. Another patient, operated upon two or three years ago, had had no complications, and had obtained complete relief from his previous symptoms.—Mr. Mole mentioned a case he had operated upon. The patient had died from pneumonia about ten days after the operation.

*January 10th, 1906.*

Mr. John Dacre, President, in the Chair.

Mr. H. F. Mole gave a demonstration on the **anatomy** and **surgical anatomy** of the **temporal bone,** illustrated by lantern slides, specimens and stereograms. This demonstration was highly appreciated by the members of the Society, and the speaker was congratulated upon its excellence by Dr. P. Watson Williams and Mr. J. Lacy Firth.

Dr. P. Watson Williams read a paper on **Why defective nasal respiration impedes growth and development.** (For a full report of this paper see p. 17 of the present issue of the *Journal.*)—Dr. E. C. Williams asked at what age the removal of adenoids gave the best results. Adenoids were met with frequently in children from the country, and it was a question whether humidity had a causal effect.—Dr. Harrison alluded to the fact that with nasal respiration one gained a better sense of having filled the chest than with mouth breathing.—Dr. Watson Williams replied that the feeling of chest-fulness alluded to was a subjective one. In his opinion infective conditions had much to do with the production of adenoids.

Mr. H. G. Kyle read notes on a case of **hydatid cyst of the liver.** The patient was a young woman. In September, 1903, a small lump which she had had for some months on the thoracic margin on the left side began to enlarge. When seen by the speaker there was a large tumour in the upper part of the left side of the abdomen. It extended down to the umbilical level and just over the middle line to the right, and moved freely with respiration. No fluctuation could be made out. The heart was displaced upwards. The long history seemed to exclude sarcoma as a diagnosis. It was found on exploration to be a large hydatid cyst in the left lobe of the liver. It was incised, and the endo-cyst removed and the fibrous capsule sutured to the parietes. During the after-treatment there was free suppuration. No daughter cysts had been seen.

Dr. J. R. Charles read a paper on **Fright in its relation to medicine.** (See p. 23 of the present issue.)—Dr. Nixon mentioned the case of a man who was liable to attacks of jaundice which he attributed to fright.—Dr. J. O. Symes expressed the opinion that many cases of chorea were directly due to fright apart from all rheumatic association, though the rheumatic child, being often of a nervous excitable temperament, was more likely to suffer than other children. He did not agree that cold played a part in the causation of rheumatic chorea. The micro-organism of rheumatic fever was least active in the coldest months.—Dr. Watson Williams thought that Graves's disease might be a secondary effect of fright. He considered that in aggravated cases the condition of the thyroid gland with hyper-activity, followed in course of time by atrophy and myxœdema, was analogous to the so-called paralytic secretion of salivary glands following section of the sympathetic. The syndrome of Graves's disease was that corresponding to the secondary effect of intense fright, and might be due to disorder of centres for emotion, higher than the somatic reflex centres in the bulb, but below the highest cortical volitional and ideational centres.—Dr. Shingleton Smith alluded to Warren's story of catalepsy being induced by a thunderstorm. A case in which a copious purpuric eruption on the upper part of the body had followed a thunderstorm was probably due to a fit having been induced by fright, and the eruption resulted from the fit.—Dr. Charles replied. He failed to see that any differentiation of cases of chorea into these due to fright and those due to rheumatism could be established.

---

*February 14th, 1906.*

Mr. John Dacre, President, in the Chair.

Dr. E. W. Hey Groves showed the following card specimens:—(*a*) **tumour of the adrenal gland,** (*b*) **parosteal sarcoma of finger,** (*c*) **primary sarcoma of thyroid,** (*d*) **sarcoma of testis,** (*e*) **duodenal ulcer** which led to fatal hemorrhage.

Dr. E. C. Williams showed a child with **hemiplegia.**

Dr. J. O. Symes read a note on **The arthritis accompanying erythema nodosum.** In the speaker's experience not more than 10 per cent. of the patients with erythema nodosum gave a history of rheumatic fever. The decades of life in which the affection was most frequently met with corresponded with those in which rheumatic fever most often occurred. The eruption was little, if at all, influenced by salicylates. Not only was the history of rheumatic fever rarely obtained in these cases, but when joint trouble was found associated with the erythema that trouble was of a different type to that of rheumatic fever.—Dr. Harrison said the subject was one in which he had taken much interest, and some years ago, from a consideration of the cases he had seen, had come to a somewhat similar conclusion as Dr. Symes. He quoted some remarks he had made on the subject in an opening address given before the Dermatological Society. In spite of a careful attempt to trace his patients he had never seen a single case in which the affection had recurred. His youngest patients had been of three and four years of age, his oldest of fifty-one and fifty-three. If you see one case you are almost sure to see others about the same time. —Dr. Kenneth Wills alluded to the relation between erythema nodosum and erythema multiforme. It had occurred to him that erythema nodosum might be classed in the group of erythemata included in erythema multiforme. Though salicylates had no influence upon the pain, he was under the impression that they shortened the duration of the disease.—Dr. F. H. Edgeworth said that he had given up the use of salicylates in the treatment of erythema nodosum. The effect of these drugs in erythema multiforme was very different. Now that the organism of rheumatic fever had been discovered it would be well worth trying to find what the organism was which caused erythema nodosum.—Dr. Carey Coombs described a case of erythema nodosum in which the joint trouble at first was unlike that of rheumatism, but later the patient had developed typical rheumatic joints.—Dr. Symes replied that he was certainly more inclined to regard erythema nodosum as a specific fever than as a mere skin affection. It often affected two or more members in a family, was associated with grave constitutional symptoms, and seemed to confer some degree of immunity.

Dr. J. J. S. Lucas read a paper on **Some points in the prognosis of pulmonary tuberculosis.** (See p. 12 of this *Journal*.—Dr. Elliott said that in treatment pure air was the important thing, but he did not think that cold and damp air was beneficial.—Dr. Shingleton Smith thought the speaker had been rather too pessimistic. The profession knew that the public expected too much from sanatorium treatment. He thought the great problem of the future was to make suitable provision for the employment of the patients who were discharged from the sanatoria.—Mr. Roger Williams alluded to the fact that he had in a large number of *post-mortems* found that 20 per cent. of the subjects presented evidences of healed tubercular processes in the lungs. In the last fifty years the mortality of phthisis had decreased threefold, and two-thirds of this decrease had preceded the discovery of the tubercle bacillus. He was inclined to attribute the improvement to better food and better sanitation, and he thought better food had had the greatest effect. Better food was the result of greater prosperity. England had a low mortality from tubercle as compared with other European countries. The permanent inhabitants of the Riviera had a high mortality from the disease, and yet we sent our patients there. The mortality decrease from phthisis had occurred during a period in which urbanisation had flourished. The speaker believed that in the future the congregation of the victims of this and other diseases would be regarded as unwise.—Dr. Leonard alluded to consumption as met with in factory hands. He had followed up cases in which young girls had been accepted at a factory with signs suggestive of incipient phthisis, and found that only a small proportion had subsequently suffered from development of the disease, and he thought that vastly more people recovered from phthisis than was generally thought.—Dr. Edgeworth thought the prognosis in phthisis was worse with basic than with apical disease, and worse with one base and one apex than with two apices diseased. He had seen one or two cases recover, though the rapidity of the pulse had been great, without the temperature accounting for it. For recovery the intestinal tract must remain healthy.—Dr. MICHELL CLARKE alluded to the great difficulty of prognosis in individual cases. If physical signs were limited to one apex the X-rays often showed there was disease in the other lung. The influence upon prognosis of a previous attack of pleurisy was worth consideration. He thought such a history might be a good point, as showing that the case would at any rate run a long course. He still thought the old idea that if the sound lung came across the sternum the case would be favourable was correct. Open-air treatment had a great effect in lessening the temperature. The pulse must be observed for long periods if correct inferences were to be drawn from it. Prognosis depended upon whether the tendency to caseation or to fibroid change was in the ascendancy.—Dr. CHARLES alluded to the effect of different diatheses upon the course of phthisis.—Dr. LUCAS replied. He believed the disease broke out again after apparent cure in the vast majority of people who had it, and belonged to the lower and middle classes. The *post-mortem* evidence of healed phthisis pointed to the disease in the cases as having been of very small extent. It was the rapid, low-tension pulse that was of bad omen.

Dr. CAREY COOMBS read a paper upon **Colitis polyposa** and showed a specimen of the disease. (See p. 31 of this *Journal*.) Dr. MICHELL CLARKE thought the disease might have resulted from infection, and might be an early stage of ulcerative colitis.—Mr. ROGER WILLIAMS suggested that diffused syphilis might have been the cause.—Dr. HARRISON said that clinically the case had been one of chronic diarrhœa. There had been no history of syphilis.—Dr. COOMBS thought if there had been a tendency to ulceration it should have become evident in the specimen, as the patient had suffered for six months. He thought that some parasitic cause had been at work.

J. LACY FIRTH.
H. F. MOLE (*Hon. Sec.*).

